# Topical Delivery of Niacinamide to Skin Using Hybrid Nanogels Enhances Photoprotection Effect

**DOI:** 10.3390/pharmaceutics13111968

**Published:** 2021-11-20

**Authors:** Renata Basto, Raquel Andrade, Cláudia Nunes, Sofia A. Costa Lima, Salette Reis

**Affiliations:** Laboratório Associado para a Química Verde (LAQV), REQUIMTE (Rede de Química e Tecnologia), Department of Chemical Sciences, Faculty of Pharmacy, University of Porto, 4050-313 Porto, Portugal; up200800307@icbas.up.pt (R.B.); id9263@alunos.uminho.pt (R.A.); cdnunes@ff.up.pt (C.N.); shreis@ff.up.pt (S.R.)

**Keywords:** in vitro release, jojoba oil, oleic acid, permeation enhancers, transethosomes, tween 80, UV radiation

## Abstract

Niacinamide (NIA) has been widely used in halting the features of ageing by acting as an antioxidant and preventing dehydration. NIA’s physicochemical properties suggest difficulties in surpassing the barrier imposed by the stratum corneum layer to reach the target in the skin. To improve cutaneous delivery of NIA, a hybrid nanogel was designed using carrageenan and polyvinylpyrrolidone polymers combined with jojoba oil as a permeation enhancer. Three different types of transethosomes were prepared by the thin-film hydration method, made distinct by the presence of either an edge activator or a permeation enhancer, to allow for a controlled delivery of NIA. Formulations were characterized by measurements of size, polydispersity index, zeta potential, encapsulation efficiency, and loading capacity, and by evaluating their chemical interactions and morphology. Skin permeation assays were performed using Franz diffusion cells. The hybrid hydrogels exhibited robust, porous, and highly aligned macrostructures, and when present, jojoba oil changed their morphology. Skin permeation studies with transethosomes-loaded hydrogels showed that nanogels per se exhibit a more controlled and enhanced permeation, in particular when jojoba oil was present in the transethosomes. These promising nanogels protected the human keratinocytes from UV radiation, and thus can be added to sunscreens or after-sun lotions to improve skin protection.

## 1. Introduction

Bioactive compounds have been delivered across the skin as an alternative to the oral route, which faces substantial challenges regarding the irregular absorption in the gastrointestinal tract, and the low bioavailability for drugs with short plasma half-life times and liver first-pass metabolism [1,2]. Topical drug administration is the term utilized for the application of the drug to specific areas of the skin for a localized effect and which is not intended for systemic distribution [3]. This administration route across the skin can offer many advantages, such as improved patient compliance, the circumvention of liver first-pass metabolism, reduced side effects associated with peaks and troughs in drug plasma concentration, the ease of dose termination when adverse effects occur, sustained drug release, and a high local concentration of the drug rather than systemic levels, thus providing a local effect [4,5].

Niacinamide (NIA), chemically known as pyridine-3-carboxamide, is an amide form of vitamin B3 and a water-soluble molecule with a wide range of mechanisms of action that have been described in the literature [6,7]. This vitamin is not naturally stored in the body, but it can be obtained by the dietary intake of vitamin B3 and tryptophan [8]. NIA is a precursor to energy co-factors in vivo, such as nicotinamide adenine dinucleotide and nicotinamide adenine dinucleotide phosphate [9,10]. Since these co-enzymes are involved in many cellular oxidation–reduction reactions, and thus can potentially influence many skin mechanisms, the intake of NIA is very important [11]. Yet, the skin provides an effective physical barrier between the external stressors and the inner system, and its barrier function mainly resides in the stratum corneum (SC), which represents the major challenge in the cutaneous administration of molecules highly dependent of their physicochemical properties [12,13,14,15,16]. Although NIA is a small molecule with a size of 122.1 Da, it is extremely soluble in water (212.95 mg/mL) and has a log *p* value of −0.37, which indicates that this bioactive compound alone may not be ideal for topical delivery [17]. Drug delivery systems emerged as a tool to overcome NIA’s physicochemical properties that hinder its penetration through the skin. Lee et al. studied the skin permeability and the anti-melanogenesis activity of NIA by incorporating it into flexible liposomes with the edge activator dipotassium glycyrrhizate, proving that the skin permeability of NIA in these flexible liposomes was significantly higher than that of the conventional liposomes [18]. Offerta and co-workers evaluated different strategies to optimize the percutaneous absorption of NIA and soy phytosterols by making use of solid lipid nanoparticles and penetration enhancers such as hydrogenated lecithin [6]. Novel microneedle-like particles were designed to disrupt the skin during the rubbing process and enhance NIA penetration [19]. Skin deposition with a low permeation of NIA will benefit the cutaneous environment, improving hydration and preventing oxidative stress and inflammation. To pursue a novel approach for the topical application of NIA, hybrid nanogels made from a combination of a hybrid hydrogel and transethosomes are explored in this study. 

Hydrogels have numerous advantages in drug delivery including increased biocompatibility, tunable biodegradability, low toxicity, proper mechanical strength, and a porous structure adequate to incorporate the active agent [20]. Given the viscoelastic and gelling properties, kappa (κ)-carrageenan (k-CRG) is one of the most commercialized seaweed-based polysaccharides for drug delivery and tissue engineering purposes [21,22,23,24]. This natural polymer closely mimics the glycosaminoglycan structure, one of the most important constituents of the native tissues’ extracellular matrix, as it comprises repeating units of (1,3)-d-glucopyranose and (1,4)-3,6-anhidro-α-d-galactopyranose with one sulfate group per disaccharide unit [25,26]. Blends between the natural and synthetic polymers lead to hybrid hydrogels, merging the advantageous properties of both. Synthetic polymers’ most wanted features are associated with their good mechanical strength, desirable flexibility, chemical inertness, and established structures over natural polymers [27]. Polyvinylpyrrolidone (PVP) is biodegradable, biocompatible, water-soluble, temperature-resistant, and pH stable with good binding properties for combination with natural polymers [28]. Even so, the hybrid hydrogels may exhibit a restricted mechanical performance and a low capability to achieve high loading and sustained drug release, mainly due to their inherently hydrophilic nature [29,30]. Hydrogels containing nanoparticles, also known as nanogels, are promising drug carriers for therapeutic applications and represent a means to overcome these limitations [31,32,33,34]. Transethosomes (TEs) merge the advantages of classic ethosomes and transferosomes, with the great capability of becoming deformable and penetrating well into the skin [35,36,37]. Since the transethosomes contain both the hydrophobic and hydrophilic entities, they can enclose the drug molecules with a wide range of solubility [38], and have edge activators or permeation enhancers (also known as surface active agents) [35,39] in their composition that lead to alterations in the organization of the *SC* lipids, thereby increasing TEs penetration through the skin [40,41]. Edge activators are membrane softening agents that are capable of changing the membrane’s flexibility and enhancing the permeability across the skin [42]. Here, different types of TEs were evaluated upon incorporation in hybrid CRG-PVP hydrogels as topical delivery systems for NIA to improve skin photoprotection. 

## 2. Materials and Methods

### 2.1. Materials and Instrumentation

Niacinamide (NIA), k-C RG, polyvinylpyrrolidone (PVP), jojoba oil, tween 80, oleic acid, 4-(2-hydroxyethyl)-1-piperazineethanesulfonic acid (HEPES) powder, k-carrageenan, chloroform, jojoba oil, trypsin, and phosphate-buffered saline (PBS) were supplied by Sigma-Aldrich (Alabaster, AL, USA). l-α-phosphatidylcholine (EPC) was purchased from Avanti Polar Lipids (Alabaster, AL, USA). Ethanol absolute (≥99.8%) and methanol (≥99.8% HPLC grade) were obtained from Fisher Chemical (Thermo Fisher Scientific, Loughborough, UK). Double-deionized water was provided by an ultra-pure water system (Arium Pro, Sartorius AG, Gottingen, Germany). The porcine ears were acquired in a local slaughterhouse (Porto, Portugal). Reagents were weighted in a digital analytical balance Kern ACJ/ACS 80-4 (Kern & Sohn; Balingen, Germany). The pH measurements were achieved using a Crison pH meter GLP 22 with a Crison 52-02 tip (Crison; Barcelona, Spain). 

### 2.2. Methods

#### 2.2.1. HPLC Analysis

NIA analysis was conducted using an HPLC equipped with a reversed-phase monolithic column (Chromolith^®^ RP-18e, 100 mm × 4.6 mm i.d., Merck) connected to a Jasco (Easton, PA, USA) HPLC system (pump PU-4180, autosampler AS-4050 and LC-Net II/ADC controller) coupled to a PDA detector (Jasco MD-4010, start wavelength = 200 nm, End Wavelength = 400 nm). The data processing was performed by ChromNAV 2.0 HPLC software (Easton, PA, USA). The chromatogram was acquired at a wavelength of 263 nm, and the retention time of NIA was at 4.8 min. The mobile phase was a mixture of water and methanol (80:20, *v*/*v*) and eluted at a flow rate of 1.0 mL/min. The injection volume was set to 20 μL with the column temperature at 30 °C. Prior to use, the mobile phase was degassed in an ultrasonic bath for 30 min. Validation of HPLC method was performed by evaluating linearity, range accuracy, precision, lower limit of quantification (LOQ), and upper limit of detection (LOD) according to established guidelines [43]. The calculated LOD was 0.18 µg/mL and the LOQ was 0.53 µg/mL.

#### 2.2.2. Preparation of Hybrid Hydrogels

Carrageenan hydrogels were prepared by dissolving the polymer powder in double-deionized water and heating at 60–70 °C while stirring in a magnetic stirring plate (IKA-Werke, Staufen, Germany) for 30 min or until a clear solution was obtained. The mixture was allowed to cool slowly during 24 h, until the gelation was complete. In hydrogels containing NIA, the active agent was added 10 min before the cooling step. Visual appearance, texture, and pH were evaluated in 24 h.

PVP was the synthetic polymer selected for the preparation of hybrid hydrogels in a ratio of 1:1 (*w*/*w*). To study the effect of a permeation enhancer on the ability of CRG hydrogels to permeate NIA through the skin, jojoba oil was selected and added to the CRG-PVP mix at the same time interval of both polymers (0.5%, 1%, 5% *w*/*v*). Hybrid hydrogels were loaded with NIA 1% (*w*/*w*) regarding the total polymer weight by incorporation at room temperature.

#### 2.2.3. Preparation of the Transethosomes Formulations

Transethosomes were prepared by the thin-film hydration method [44]. Three types of TEs were prepared with different surface-active agents (tween 80, oleic acid, and jojoba oil) and their composition is shown in Table 1. Briefly, EPC and surface-active agents were weighed and dissolved in a mixture of methanol: chloroform (1:3, *v*/*v*). By maintaining pressure under vacuum for 20 min, the organic phase was slowly evaporated at 40 °C using a rotary evaporator (Buchi Rotavapor R-200, Switzerland) until a thin clear film of lipid mixture was formed. The film was hydrated with water for non-loaded TEs and a water solution containing 30 mg of NIA for loaded-TEs. Formulations were vortexed for 2 min and, when necessary, the water bath at 45 °C was used to enhance the detachment of the thin film. The specified volume of ethanol was added, and the formulation was vortexed for 1 min. All prepared dispersions were subjected to ultra-sonication using a probe sonicator (VCX130, Sonics and Material Vibra-CellTM with a CV-18 probe; 115 Newtown, CT, USA) for 5, 10, and 30 s regarding the TE tween 80, TE oleic acid, and TE jojoba oil, respectively. All products were kept at 4 °C until use per se or for incorporation within the CRG-PVP hydrogels.

#### 2.2.4. Fourier-Transform Infrared Spectroscopy Evaluation

About 3 g of each hydrogel and 2 mL of each TE were placed into separate 15 mL falcons and kept overnight in a −80 °C freezer (Deep Freezer, GFL^®^, Burgwedel, Germany). Then, the lyophilization proceeded for 72 h in a freeze drier (LyoQuest –85 plus v.407, Telstar^®^ Life Science Solutions, Terrassa, Spain) at −80 °C and 0.40 mbar of pressure. The freeze-dried hydrogels and TEs were analyzed using a FTIR spectrophotometer (Frontier^TM^, PerkinElmer; Santa Clara, CA, USA) equipped with an attenuated total reflectance device. Obtained spectra were a result of 32 combined scans recorded between 4000 and 600 cm^−1^, with spectral resolution of 8 cm^−1^.

#### 2.2.5. Determination of Loading Capacity

The ultrafiltration method was utilized for the estimation of drug loading capacity of developed formulations, using an Allegra^®^X-15R centrifuge (Beckman Coulter, Pasadena, CA, USA). TEs containing NIA were diluted 10× with methanol and placed in clean Amicon Ultra-4 centrifugal filter units (Merck Millipore Ltd., Carrigtwohill, Co Cork, Ireland). These devices were centrifuged at 2250× *g* for approximately 30 min, or until complete separation between the TEs retained in the filter and the solvent phase corresponding to the supernatant. The supernatant representing the unentrapped NIA was rejected and the filter portion of the Amicon tubes were put upside down inside a 50 mL falcon tube and centrifuged at 2250× *g* for 15 min, until the pellet was completely transferred. The sediment was lysed using methanol and water was added to match the ratio of the mobile phase used in the quantification method. The amount of NIA in the pellet was quantified using HPLC analysis, as previously described. 

The loading capacity (*LC*) was calculated by the following formula:

(1) $$LC\;\left(\%\right)=\frac{Total\;entrapped\;drug\;mass}{Total\;TE\;mass\;\left(lipid+surface\;active\;agents\right)}\times 100$$

#### 2.2.6. Morphology Evaluation

##### Scanning Electron Microscopy

Freeze-dried hydrogels were fixed onto carbon-taped metal pins. Prior analysis by scanning electron microscopy (SEM) using a FEI Quanta 400 FEG ESEM/EDAX Pegasus X4M at an accelerating voltage of 10 kV, the pins were coated with Au/Pd by sputtering for 45 s.

##### Transmission Electron Microscopy

TEs samples were dropped on a copper grid and left for 1 min. After removing the excess with filter paper, 10 µL of 0.75% (*w*/*v*) uranyl acetate was added for 30 s. Then, the TEs morphology was observed by transmission electron microscopy (TEM, Jeol JEM-1400; JEOL, Ltd., Tokyo, Japan) at the accelerating voltage of 60 kV.

#### 2.2.7. Evaluation of the Physicochemical Properties of Transethosomes 

The physicochemical properties of TEs were assessed with a ZetaPALS zeta potential analyzer (Brookhaven Instruments Corporation; Holtsville, NY, USA). Particle size (PS), polydispersity index (PDI), and zeta potential (ZP) were obtained after 5× times dilution in water. For particle size measurements, six runs of 2 min were performed at room temperature while for zeta potential six runs of 10 cycles were performed at a scattering angle of 90°.

#### 2.2.8. Stability Studies

Storage stability of TEs was evaluated at 4 °C. Samples were withdrawn each week and stability was evaluated by comparing the initial physicochemical parameters with results obtained after storage. After 2 weeks, a significant loss of NIA was detected.

#### 2.2.9. In Vitro Drug Release Assay

To evaluate the release of NIA from the NIA-loaded TEs, in vitro release assays were performed using Slide-A-Lyzer^®^ mini dialysis device containing a cellulose membrane. Briefly, 200 µL of NIA-loaded TEs (TE tween 80, TE oleic acid and TE jojoba oil TEs) and 250 µL of free NIA solution (to a final mass of 50 µg) were pipetted to the devices and placed in beakers containing 20 mL of HEPES buffer (pH = 7.4). HEPES buffer was kept at 37 °C for 6 h under constant stirring, and 1 mL of medium was taken at timepoints 0.5, 1, 2, 3, 4, 5 and 6 h. The receptor phase was immediately replenished with an equal volume of fresh receptor medium.

#### 2.2.10. Skin Permeation Studies

Skin permeation studies were performed using a Franz diffusion cell system (9 mm unjacketed Franz diffusion cell with 5 mL receptor, O-ring joint, clear glass, clamp, and stir-bar; PermeGear, Inc., Hellertown, PA, USA). The control sample in all experiments was free NIA dissolved in water at the same amount as in all the formulation samples. Skin samples were obtained from pig ear skin of healthy animals at a local slaughterhouse, with no ethical approval needed. The full thickness membranes obtained were stored at −20 °C for no longer than two weeks before use. Skin samples were fixed between the donor and the receptor phase, with SC facing upwards into the donor compartment. HEPES buffer (pH 7.4) was used as the receptor medium to allow the establishment of physiological mimetic conditions and to sustain permeant solubilization, stirred and thermostated at 37 ± 1 °C, during all experiments. The sample was applied to the skin surface (0.636 cm^2^) of the donor, and 1 mL of the receptor phase was withdrawn through the sampling port of the Franz diffusion cell at defined intervals (1, 3 and 6 h). The receptor phase was immediately replenished with an equal volume of fresh receptor phase. The withdrawn sample was analyzed by HPLC, and the amount of NIA was determined using the previously described conditions. TEs were filtered by the ultrafiltration method before the experiments to separate the non-retained NIA from the loaded-TEs. 

To quantify the amount of NIA retained in the skin, skin digestion studies were performed using the Ultra-Turrax at 1200 rpm for 2 min or until complete destruction of skin samples in 5 mL of previously described mobile phase. The falcons containing skin samples were placed in an ultrasound bath for 45 min to help extract the bioactive compound. Following this step, samples were centrifuged for 20 min at 2250× *g* to pellet the skin remains, and the supernatant was collected and placed in a glass tube for evaporation in the rotary vapor. The dried sample was resuspended in 1 mL of mobile phase with the help of vortex and, when necessary, the water bath was used to enhance the detachment of the thin film. The amount of NIA deposited was quantified by HPLC analysis using the previously described conditions. 

Briefly, the apparent permeability (P_app_) was estimated by the ratio of the sum of the mass of NIA (m_a_/g) permeated across membranes and the product of the initial mass in donor chamber (m_d_/g), the surface area of the skin (A = 0.636 cm^2^), and the time (t = 3, h = 10,800 s), following the equation:

(2) $$P_{\mathrm{app}}\;\left(\mathrm{cm}/s\right)=\frac{\sum m_{a}}{m_{d}\cdot A\cdot t}$$

#### 2.2.11. Cellular Viability Assays

To determine the effect of the unloaded and NIA-loaded hydrogels and nanogels on human keratinocytes (HaCaT cells), a cellular viability assay was performed through assessment of (3-(4,5-dimethylthiazol-2-yl)-2,5-diphenyltetrazolium bromide) tetrazolium reduction assay) metabolic activity (MTT assay). HaCaT cells were cultivated in Dulbecco’s modified Eagle medium (DMEM) media supplemented with 10% (*v*/*v*) fetal bovine, 1% (*v*/*v*) streptomycin/penicillin and 1% (*v*/*v*) amphotericin B, and maintained at 37 °C in a humidified atmosphere of 5% (*v*/*v*) CO_2_. The cell viability assay was conducted in 96-well plates containing 4 × 10^4^ cells per well. After cell adhesion, the culture medium was replaced by serial dilutions (2.5 to 200 µM) of free NIA, hydrogel with and without NIA, and NIA-loaded nanogels. After 24 h of exposure, the supernatant was removed and the MTT solution (0.5 mg/mL) added for 2 h prior replacement with dimethyl sulfoxide. Then, the absorbance was measured at 590 nm using a Synergy^TM^ HT Multi-Mode Microplate Reader (BioTek Instruments Inc., Winooski, VT, USA), subtracting the background read at 630 nm. Cells grown only in culture medium and cells treated with Triton^TM^ X-100 (1% *v*/*v*) were used as positive and negative controls, respectively. Results were expressed as a percentage of metabolic activity relative to the values obtained for non-treated cells. 

#### 2.2.12. Ultraviolet B Irradiation–Photoprotective Assay

HaCaT cells were cultivated at 4 × 10^4^ cells per well in 96-well plates, as previously described. At first, 3 ultraviolet B (UVB) intensities were evaluated (80, 100 and 150 mJ/cm^2^) to determine the most suitable to reduce cell viability in about 50%. An amount of 80 mJ/cm^2^ was selected for the study. Prior to irradiation, cells were treated with 100 mM of NIA as free solution, hydrogel, and nanogel for 4 h. Empty nanogels were used as controls as well as non-irradiated cells. To prevent UVB light absorption by the cell culture medium, the medium was replaced by a thin layer of phosphate buffer solution to cover the cells during irradiation. The irradiation step was conducted using an illuminator system with UVB lamp (TRIWOOD 31/36, Helios Italquartz, Milan, Italy) for 80 s. The intensity of the lamps was verified by an HD 2302.0 radiometer (Delta OHM, Padova, Italy). Then, the cells were incubated in fresh culture media for 24 h prior to cell viability assessment using MTT assay, as described above.

#### 2.2.13. Statistical Analysis

Statistical analysis was performed using the GraphPad Prism software (Version 6.01 for Windows; GraphPad Software Inc., San Diego, CA, USA). The one-way and two-way ANOVA analyses of variance were used to assess the differences between formulations and a value of *p* < 0.05 was considered statistically significant.

## 3. Results and Discussion

### 3.1. Preparation and Characterization of Niacinamide-Loaded Hybrid Hydrogels

Aiming to explore the advantages of the natural and synthetic polymer properties, CRG and PVP combinations were pursued. Hybrid hydrogels were prepared from an aqueous polymeric blend consisting of CRG and PVP, both of which are biocompatible polymers. Vegetable oils have been found to be effective penetration enhancers due to the presence of fatty acids; thus, jojoba oil was the natural oil selected for the hydrogel formulation [45]. The effect of the jojoba oil amount in the hybrid hydrogel was evaluated. A quantity of 5% (*w*/*v*) jojoba oil is excessive, leading to the saturation of the mixture and the immiscibility of the oil with the two polymers (Figure S1). So, the assessment proceeds with 0.5 and 1% of jojoba oil in the hydrogels.

**Figure 1:**
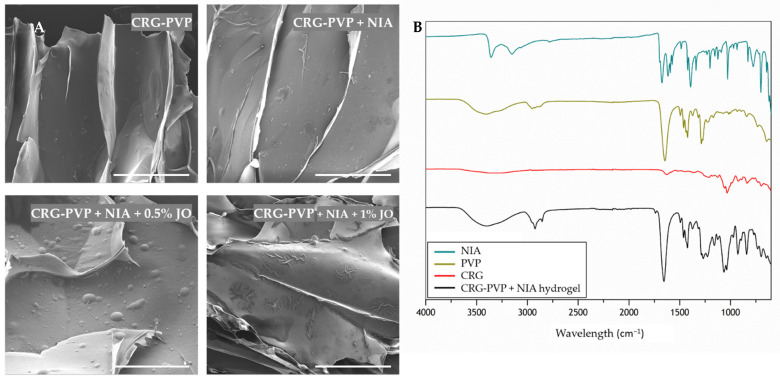
Analysis of the hydrogels morphology (**A**) and chemical interactions (**B**). SEM of CRG-PVP hydrogel matrix with NIA and jojoba oil in different amounts (0.5% and 1%). Scale bar 200 µm, amplification of 500×. FTIR spectra of CRG-PVP NIA-loaded hydrogel and all reference compounds in relation to transmittance.

The SEM images of the CRG-PVP hydrogel matrix with NIA and different ratios of jojoba oil depicting the morphology, distribution, and alignment, are shown in Figure 1. The CRG-PVP hydrogel presents a three-dimensional robust macrostructure. This observation indicates that a proper combination of CRG and PVP in the hydrogel resulted in the formation of intertwined copolymer hydrogel with a highly linear and aligned network of porous channels. Yacob and collaborators showed that the mixture of CRG and PVP resulted in the formation of a hydrogel with a more regular and porous structure as compared to the structure of the hydrogel of each component alone [46]. Researchers have proven that PVP has strengthened the gel and helped in producing a more regular, compact, and porous network, as CRG alone produced a hydrogel with a non-defined structure. The incorporation of NIA in the CRG-PVP hydrogel does not appear to alter the original aligned alveolar macrostructure. Jojoba oil also does not seem to influence the size and shape of the porous co-polymer hydrogel structure; however, its presence is clear, as it appears to be deposited in the form of a smooth coating, leading to the formation of bubbles on the surface. As the amount of jojoba oil increases in the composition, the oil appears to adopt a patch shape on top of the hydrogel surface. It is noticeable the high affinity that this essential oil has for the polymer matrix, as its accumulation is visible in the area where the alveoli walls merge.

To provide evidence for the interaction of the two polymers and the incorporation of NIA upon the hydrogel production, infrared spectroscopy was performed. The characteristic peaks of the analyzed compounds are detailed in Figure 1, and spectra are given in the range 600–4000 cm^−1^. From the FTIR data analysis, it is possible to monitor the presence of CRG, PVP and NIA in the hydrogel matrix The typical spectrum of CRG, with the characteristic peaks at 1034, 922 and 836 cm^−1^ (C–O–C stretching, C–O–C of 3,6-anhydrous galactose and C–H rocking, respectively), can be observed [47]. The characteristic peaks of PVP are present at 3404, 2924, 2894 and 1646 cm^−1^ (C–O stretching, asymmetric CH_2_ stretching of pyrrole ring, symmetric chain CH_2_ stretching, and C=O stretching, respectively) [48]. In the FTIR spectrum of the CRG-PVP hydrogel, the bands corresponding to the major functional groups belonging to both components can be seen. These results indicate that the synthetic polymer PVP is miscible with the natural polymer CRG. It has been shown that the frequency of C=O stretch is very sensitive to hydrogen bond formation with water molecules [49]. The pyrrolidone rings in PVP contain a proton accepting carbonyl moiety, whereas CRG presents hydroxyl as a hydrogen donor [49]. Therefore, hydrogen bonding interactions may take place between these two chemical moieties in blends of CRG and PVP. As for NIA, the characteristic peaks are evidenced at 3358–3150, 1674, 1394 and 1028 cm^−1^ (asymmetric and symmetric NH_2_, C=O, C–N, and pyridine stretching, respectively) [50]. The hydrogel is composed of 1% (*w*/*w*) of NIA, thus it is important to note that NIA may have to bind in a higher quantity to be quantified by FTIR analysis. However, it may be that the peak at 1038 cm^−1^ in the hydrogel spectrum is representative of the NIA characteristic peak at 1028 cm^−1^, shifted due to vibrational changes from interactions with the polymers. In addition, the peak at 1654 cm^−1^ in the hydrogel spectrum may be the result of the peak of NIA at 1674 cm^−1^ masked with the peak of PVP at 1646 cm^−1^. The chemical nature of the NIA-loaded hydrogel is kept regardless of the different jojoba oil percentage in the matrix, proven by the coincidence of the FTIR curves in the position of the characteristic frequencies (Figure S2).

### 3.2. Production and Characterization of Niacinamide-Loaded Transethosomes

Aiming to explore TEs as a novel approach for the topical application of NIA, three different types of TEs varying in composition due to the presence of either an edge activator, tween 80 (TE tween 80), or a permeation enhancer, oleic acid (TE oleic acid), and jojoba oil (TE jojoba oil), were pursued. While tween 80 and oleic acid have already been studied in compositions for TEs, jojoba oil is an innovative approach and was selected not only for its composition in fatty acids, known for being good permeation enhancers, but also to evaluate its effect on TEs, since its influence was studied in the developed CRG-PVP hydrogels [45]. 

Empty TEs presented particle sizes between 133 and 320 nm, suitable for topical delivery (Table S1) [51]. Zeta potential values were determined as -15 ± 3, −31 ± 1 and −19 ± 1 mV for TE tween 80, TE oleic acid and TE jojoba oil, respectively, indicating the need to verify the stability during storage. It is well established that when the surface values are equal to or higher than |30| mV, the nanoparticles have electrostatic stabilization and a low tendency to aggregate [52]. Table 2 shows that the incorporation of NIA in the TEs did not affect the particle size. All the NIA-loaded TEs exhibited a fairly narrow size distribution and good dispersion (PDI value ≤ 0.3). The surface potential varied considerably between −17 ± 4 mV for TE tween 80 and −40 ± 2 mV for TE oleic acid. These results are expected since tween 80 is a non-ionic surfactant, resulting in lower zeta potential magnitude values [53,54], while the high zeta potential value for oleic acid TEs is due to its negative charge in a water environment. Transethosomes showed loading capacity values ranging from 5.3 to 7.6%, with TE jojoba oil being the most favorable formulation.

**Figure 2:**
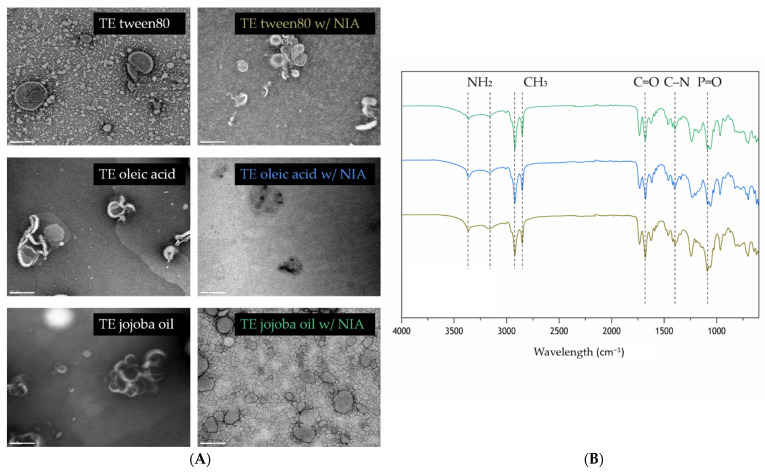
Analysis of NIA-loaded and non-loaded TEs morphology (**A**) and chemical interactions of NIA-loaded TEs (**B**). TEM of different types of TEs, with and without NIA. Scale bar 200 nm, amplification of 100,000×. FTIR spectra of TE tween 80 w/NIA (bottom), TE oleic acid w/NIA (middle), and TE jojoba oil w/NIA (top) in relation to transmittance.

The morphology of the TEs was assessed by using TEM, and the results are displayed in Figure 2. Overall, all types of TEs exhibited an almost spherical shape, which is the typical liposome structure [55]. As expected, since NIA is a small bioactive compound, its incorporation did not affect the shape nor the size of the nanoparticle. Moreover, in TE oleic acid, the surface active agent may be the compound forming the wire-like structures that can be observed in the lipid bilayer zone. When the aggregations are visible, the spherical shape becomes more irregular, which emphasizes the high deformability of the TEs membrane that enables them to adapt its form to the surrounding space [56]. However, it is possible to observe from the images that TE jojoba oil is more predisposed to the formation of aggregates in comparison with the other types.

The main characteristic molecular vibrations of the EPC transethosomes are kept, regardless of the presence of an edge activator, tween 80, or a permeation enhancer like oleic acid and jojoba oil (Figure 2). Storage stability was evaluated in terms of particle size, PDI, and zeta potential over 2 weeks at 4 °C temperature. Collected data remained practically constant (Figure S3); however, variations were observed for EE% and LC% values after 2 weeks. Overall, the formulations composed of oleic acid are less stable compared to the other two types, given the observed reduction of EE% and LC% values by half (Figure S4).

**Figure 3:**
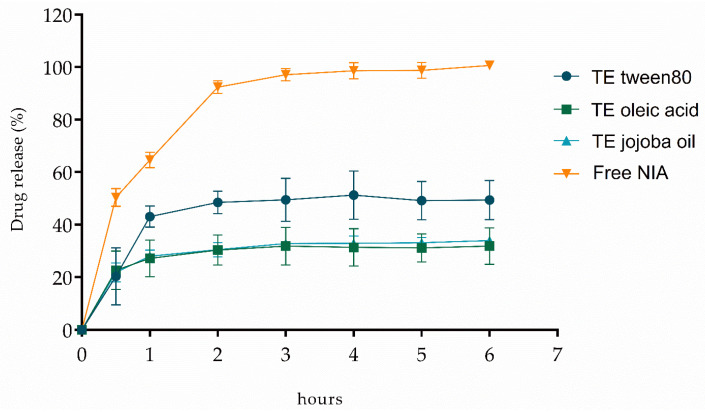
Drug release profile of NIA as free drug and loaded in tween 80, oleic acid, and jojoba oil transethosomes. Each value represents the mean ± SD (*n* ≥ 2).

The in vitro release of NIA from the different types of TEs was evaluated for 6 h. Figure 3 shows that free NIA has the highest release percentages over time, as expected for a non-encapsulated drug. A burst release is denoted up to 2 h, then reaches stabilization, indicating that after 6 h, all free NIA passed through the membrane. On the other hand, the encapsulated NIA leads to lower release values, highlighting the potential of the sustained release from TEs. TEs tween 80 are slightly faster at releasing NIA compared with the other two types, stabilizing at 50% of release. Oleic acid TEs and jojoba oil TEs have a similar release profile that stabilizes at 32% and 34% of released NIA, respectively. This indicates that tween 80 has a lower retention effect on NIA than oleic acid and jojoba oil.

### 3.3. Nanogels for Niacinamide Topical Delivery

The innovative combination of these two drug delivery systems was thought to evaluate the possible permeability enhancements from the hydrogel and/or the transethosome. To determine the skin permeability of the TEs-loaded hydrogels, the CRG-PVP hydrogel w/0.5% jojoba oil was selected to incorporate the NIA-loaded TEs (tween 80, oleic acid and jojoba oil), forming the nanogels. The choice of the hydrogel was based not only on the respective in vitro permeation assays (Figure S5) but also on the amount of jojoba oil (0.5%). 

**Figure 4:**
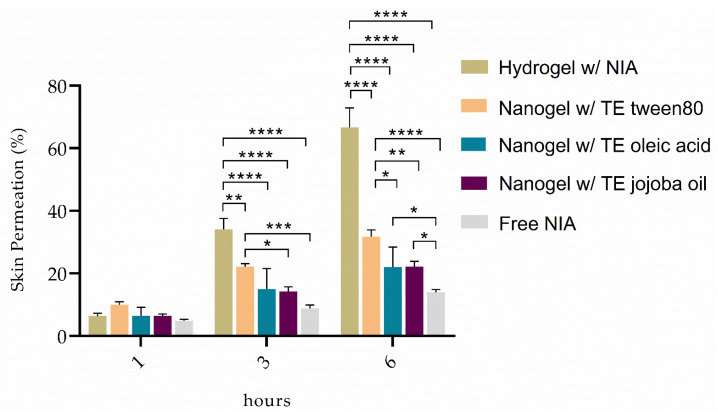
In vitro skin permeation results of free NIA, NIA-loaded hydrogel, and NIA-loaded nanogels (TE tween 80, oleic acid, and jojoba oil) from three independent experiments (*n* = 3). Asterisks indicate statistical significance (*, *p* ≤ 0.05; **, *p* ≤ 0.01; ***, *p* ≤ 0.001; ****, *p* ≤ 0.0001). No asterisks indicate no statistical significance (*p* > 0.05).

From Figure 4, it can be seen that the NIA-loaded hydrogels lead to higher drug permeation compared to the NIA-loaded nanogels. After 1 h, although the differences are not significant, there is a certain prominence of the hydrogel containing TE tween 80, whose permeation rate slowly increases over time, being surpassed by the permeation capacity of the hydrogel alone. Nevertheless, the NIA-loaded TE tween 80 hydrogel capacity to permeate NIA is always superior to the other two types of TEs incorporated within hydrogel. The combination of hydrogel with TE jojoba oil appears to hamper the ability to permeate NIA, which may be due to the fact that both delivery systems have a certain amount of jojoba oil, resulting in a greater retention of NIA in the upper layers of the skin, which is favorable for skin photoprotection. The skin digestion results obtained are in agreement with the data from the permeation assays, as shown in Table 3. Free NIA showed the lowest deposition value (6.5 ± 0.8%) among all the formulations, leading to the conclusion that the majority of the bioactive remained in the apical compartment. This result emphasizes the expected difficulty of NIA to penetrate and permeate the skin layers, as already reported by other research groups [57,58]. A greater retention of NIA in the skin layers can be verified in the case of the nanogels containing TE jojoba oil, which showed a more controlled permeation among the nanogels. 

Table 4 summarizes the apparent permeability coefficients of the free NIA and NIA-loaded hydrogels and nanogels developed in this study, to analyze the apparent permeability rate (P_app_) of the compound in the receiver compartment, at time point 3 h. As expected, these results are in accordance with the values obtained from the permeation assays performed. Comparing to free NIA data, it is clear that all formulations have a more controlled permeation capacity, as the appearance rate of NIA is less than half in all cases. Among all formulations, it is clear that TEs are more effective at retaining NIA in the skin layers for the intended topical effect. However, due to their viscosity, they cannot be applied alone. Given the consistency and storage stability of the TEs incorporated within the CRG-PVP hydrogels, and the amount of permeated NIA and the P_app_ values, the combination of the hydrogel with TE jojoba oil is the best option for a local skin action and was further assessed in terms of the photoprotective effect in human keratinocytes.

**Figure 5:**
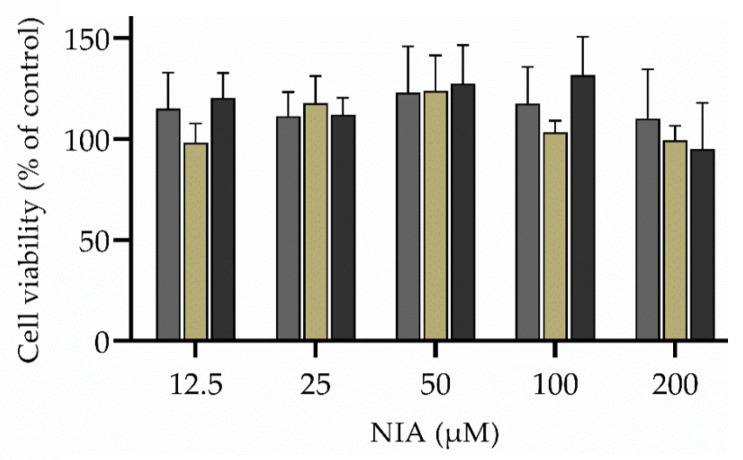
Effects NIA on cell viability in HaCaT keratinocytes. Free NIA (grey bar), NIA-loaded hydrogel (dark yellow), and NIA-loaded nanogel (dark grey) on cell viability in HaCaT keratinocytes. Each value represents the mean ± SD (*n* ≥ 3). No asterisks indicate no statistical significance (*p* > 0.05).

To investigate the biocompatibility of the developed NIA-loaded hydrogel and NIA-loaded nanogel (TE jojoba oil), HaCaT cells were first treated with various concentrations of free and loaded NIA (12.5–200 µM) for 24 h, and the cell viability was determined. The free and loaded-NIA did not exhibit any significant cytotoxicity and the cell viability was higher than 100% (Figure 5). Based on these results, the concentration of 100 µM was chosen for further cellular studies. 

**Figure 6:**
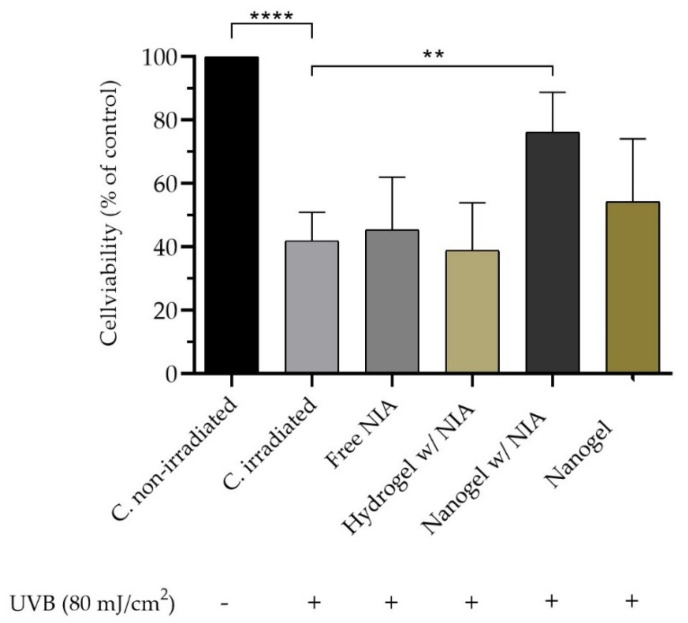
Effects of free NIA and NIA-loaded hydrogel and nanogels on cell viability in UVB (80 mJ/cm^2^) irradiated HaCaT keratinocytes at a concentration of 100 µM. Data are representative of five independent experiments as mean ± SD. Symbol – represents “absence of” while + means “exposure to”. Asterisks indicate statistical significance (**, *p* ≤ 0.01; ****, *p* ≤ 0.0001). No asterisks indicate no statistical significance (*p* > 0.05).

Human skin keratinocytes are essential cells in the skin and connective tissue and one of the major targets of UV irradiation [59]. A preliminary study evaluated the effect of different UVB intensities (80, 100 and 150 mJ/cm^2^) on HaCaT cells’ death. The intensity of 80 mJ/cm^2^ lead to ca. 50% cell survival and was statistically different from the non-irradiated control group (Figure 6). The free NIA and the NIA-loaded hydrogel did not show significant differences in cell viability in comparison to the irradiated control. However, following treatment with the NIA-loaded nanogel/TE jojoba oil, the cell viability increased up to 80%, highlighting the protection factor of this formulation. These results suggest that the protective effect may be due to the ability of TEs to penetrate skin cells and allow a localized NIA free scavenging activity. A protective effect of NIA on induced oxidative damage in human HaCaT keratinocytes was associated with the inhibition of ROS generation, lower excessive intracellular Ca^2+^, balancing membrane potential, suppressing apoptosis, and rescuing cells from lipid oxidation, protein carbonylation, and DNA damage [60]. Rodriguez-Luna and co-authors reported similar UVB protection for HaCaT keratinocytes in the presence of fucoxanthin and rosmarinic acid [61]. NIA is known to repair the oxidative stress and photolesions induced by UV irradiation, as it plays a key role in the cellular energy metabolism by being a precursor of NAD and providing energy to the irradiated cells [62]. In accordance with these properties, the combination of transethosomes containing NIA with a hybrid hydrogel produced a natural tool for the enhanced deposition of NIA in the skin, allowing for an effective photoprotective effect against skin UVB-induced disorders.

## 4. Conclusions

NIA formulated for topical application has shown several beneficial results on skin health and protection. In this study, the topical delivery of NIA mediated by CRG-PVP hydrogels resulted in less than 10% retention in the skin, which is the target tissue for slowing down ageing. As most of the applied NIA in the skin is lost, a combined approach was pursed by taking advantage of the properties of transethosomes. Given the particular composition of TEs, this type of liposome can become deformable and penetrate easily into the skin. Within the three surface active agents examined here in the TEs, the natural permeation enhancer jojoba oil exhibited the most favorable drug loading and release features. The TEs combined with the CRG-PVP hydrogels (nanogels) successfully improve NIA skin deposition with reduced permeation (1.17 ± 0.17 × 10^−5^ cm/s in comparison to 3.01 ± 0.66 × 10^−5^ cm/s, obtained for hydrogels without TEs). Some drug delivery systems have already been designed for NIA’s skin delivery, namely liposomes, solid lipid nanoparticles, and even microneedles. These focus on the improvement of skin permeability, while here the hybrid nanogels aim to retain NIA in the skin with low systemic distribution, as evidenced by the skin permeation data. These advantages from the hybrid nanogels will allow for further applications aiming a local action of the drug. Yet, more research is needed to improve the drug loading and storage stability of the TEs. In vitro photoprotection to UVB radiation in human keratinocytes was observed for the treatment of empty nanogels and NIA-loaded nanogels, indicating a possible synergistic effect of the TEs composition and NIA. The evaluation of the developed hybrid nanogels in human skin could be further pursue given their biocompatible properties and the promising delivery of NIA. Overall, the new nanogel can be further applied in sunscreens or cosmetic products to improve skin protection towards photo-aging, skin inflammation and even pre-cancerous skin lesions.

## Figures and Tables

**Table 1:** The composition of transethosomes.

Ingredient (mg)	TE Tween 80	TE Oleic Acid	TE Jojoba Oil
EPC	20	20	20
Tween 80	2	—	—
Oleic Acid	—	2	—
Jojoba oil	—	—	2
Ethanol (µL)	600	600	600
Water (µL)	2400	2400	2400

**Table 2:** Characterization of NIA loaded-TEs TE tween 80, II and III.

	PS (nm)	PDI	ZP (mV)	LC (%)
TE tween 80	132 ± 9	0.295 ± 0.006	−17 ± 4	5.3 ± 1.2
TE oleic acid	171 ± 18	0.296 ± 0.020	−40 ± 2	6.7 ± 2.2
TE jojoba oil	215 ± 6	0.232 ± 0.010	−21 ± 6	7.6 ± 2.5

Each value represents the mean ± SD of 3 independent measurements (*n* = 3). PS—Particular size; PDI—Polydispersity index; ZP—Zeta potential; LC—Loading capacity.

**Table 3:** Skin deposition of niacinamide.

	Deposition %
Free NIA	6.5 ± 0.8
NIA-loaded hydrogel	9.2 ± 0.6
NIA-loaded nanogel (TE tween 80)	10.9 ± 0.7
NIA-loaded nanogel (TE oleic acid)	27.8 ± 3.5
NIA-loaded nanogel (TE jojoba oil)	32.5 ± 2.3

Each value represents the mean ± SD of 4 independent measurements.

**Table 4:** Apparent permeability coefficient of free NIA and NIA-loaded hydrogels and nanogels.

Composition	P_app_ (×10^−5^ cm/s)
Free NIA	6.49 ± 0.99
NIA-loaded hydrogel	3.01 ± 0.66
NIA-loaded nanogel (TE tween 80)	2.58 ± 0.87
NIA-loaded nanogel (TE oleic acid)	0.90 ± 0.81
NIA-loaded nanogel (TE jojoba oil)	1.17 ± 0.17

Each value represents the mean ± SD of 4 independent measurements for hydrogels (*n* = 4) and 3 independent measurements for nanogels (*n* = 3).

## Data Availability

On request.

## Supplementary Materials

The following are available online at https://www.mdpi.com/article/10.3390/pharmaceutics13111968/s1, Figure S1: Photograph of one of the optimized hydrogel combinations (CRG-PVP (1:1) + NIA 1% (*w*/*w*) + jojoba oil 5%); Figure S2: Evaluation of jojoba oil effect on NIA-loaded hydrogel’s chemical nature through FTIR; Figure S3: Storage stability of NIA-loaded formulations at 4 °C over 2 weeks. Size, zeta potential, and polydispersity index values displayed (Size: SD < 2%; ZP: SD < 5%; PDI: SD < 3%); Figure S4: Storage stability of NIA-loaded formulations at 4 °C over 8 weeks. EE% and LC% values displayed; Figure S5: In vitro skin permeation results of free NIA and NIA-loaded developed hydrogels from four independent experiments (*n* = 4). Asterisks indicate statistical significance (*, *p* ≤ 0.05; **, *p* ≤ 0.01; ***, *p* ≤ 0.001; ****, *p* ≤ 0.0001). No asterisks indicate no statistical significance (*p* > 0.05); Table S1: Characterization of non-loaded TEs TE tween 80, oleic acid and jojoba oil.
