# Supplementary material for: Topical Delivery of Niacinamide to Skin Using Hybrid Nanogels Enhances Photoprotection Effect
Source: Pharmaceutics. 2021 Nov 20;13(11):1968. doi: 10.3390/pharmaceutics13111968 (PMC8622650; doi:10.3390/pharmaceutics13111968)
Supplement: Supplementary file 1 [file pharmaceutics-13-01968-s001.zip › pharmaceutics-1451253-supplementary.pdf]

pharmaceutics

MDPI

Supplementary Materials: Topical Delivery of Niacinamide to Skin Using Hybrid Nanogels Enhances Photoprotection Effect

Renata Basto, Raquel Andrade, Cláudia Nunes, Sofia Lima * and Salette Reis

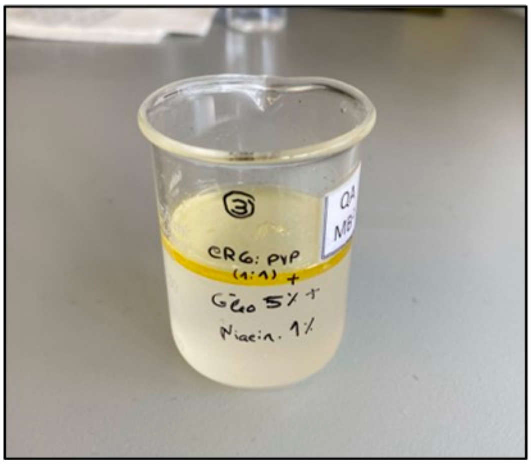

Figure S1. Photograph of one of the optimized hydrogel combinations (CRG-PVP (1:1) + NIA 1% (*w/w*) + jojoba oil 5%).

Copyright: © 2021 by the authors. Licensee MDPI, Basel, Switzerland. This article is an open access article distributed under the terms and conditions of the Creative Commons Attribution (CC BY) license (https://creativecommons.org/licenses/by/4.0/).

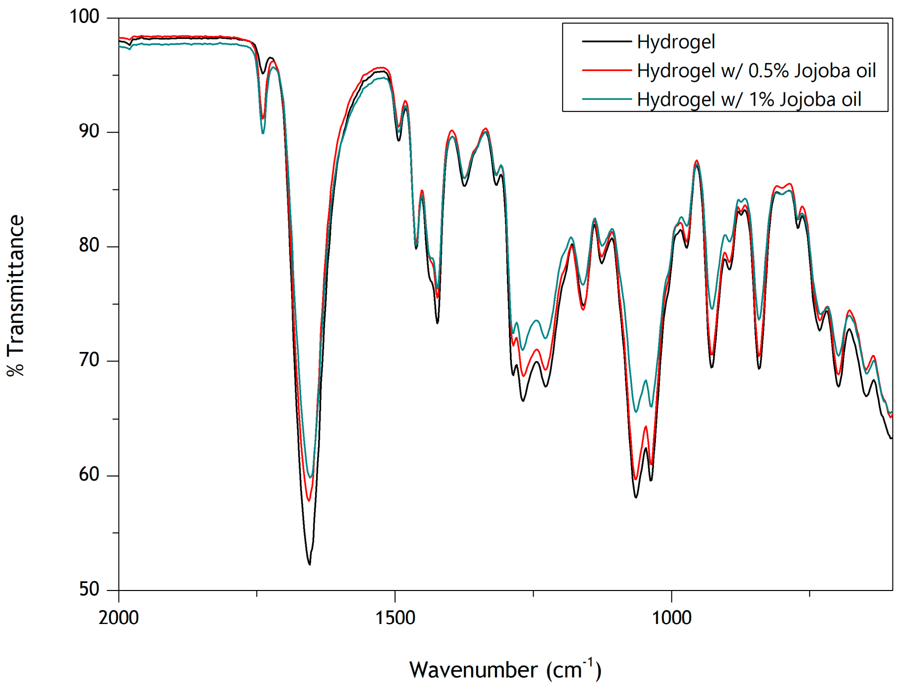

Figure S2. Evaluation of jojoba oil effect on NIA-loaded hydrogel's chemical nature through FTIR.

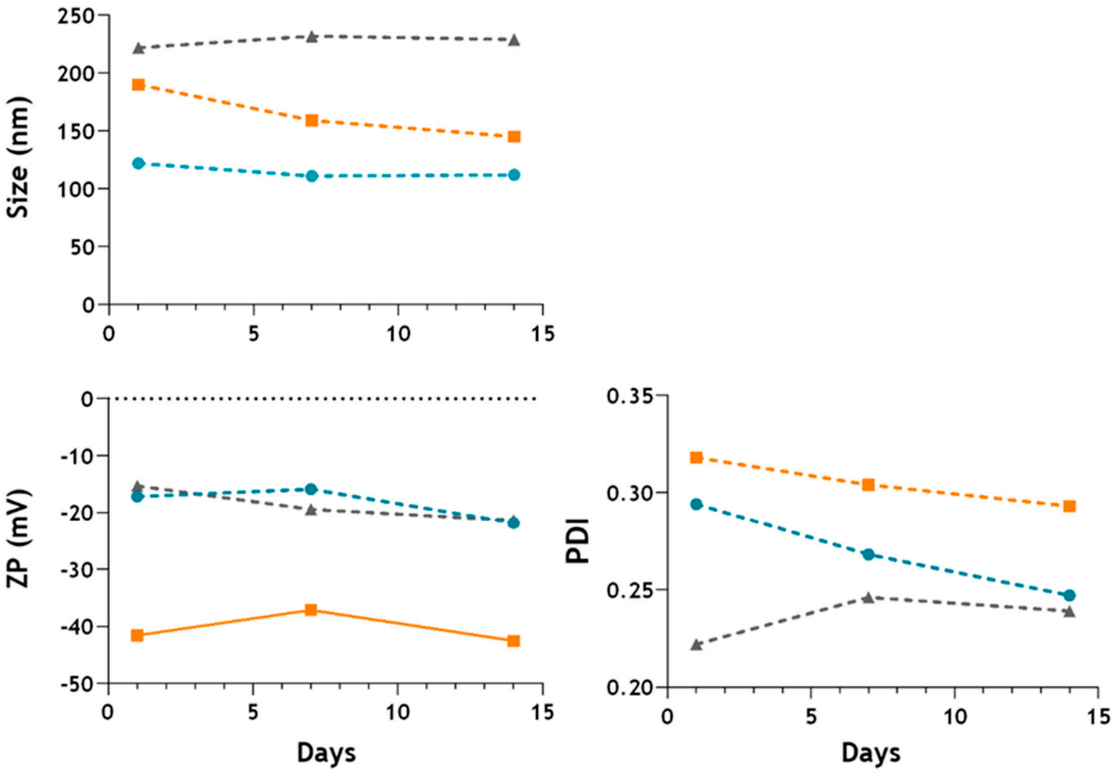

Figure S3. Storage stability of NIA-loaded TEs tween 80 (blue), oleic acid (orange) and jojoba oil (grey) at 4 °C over 2 weeks. Size, zeta potential and polydispersity index values displayed (Size: SD < 2%; ZP: SD < 5%; PDI: SD < 3%).

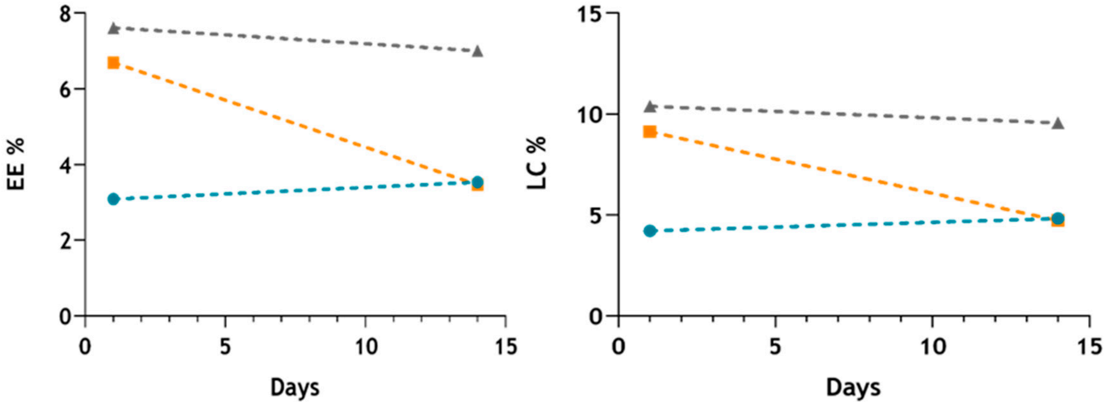

Figure S4. Storage stability of NIA-loaded TEs tween 80 (blue), oleic acid (orange) and jojoba oil (grey) at 4 °C over 8 weeks. EE% and LC% values displayed.

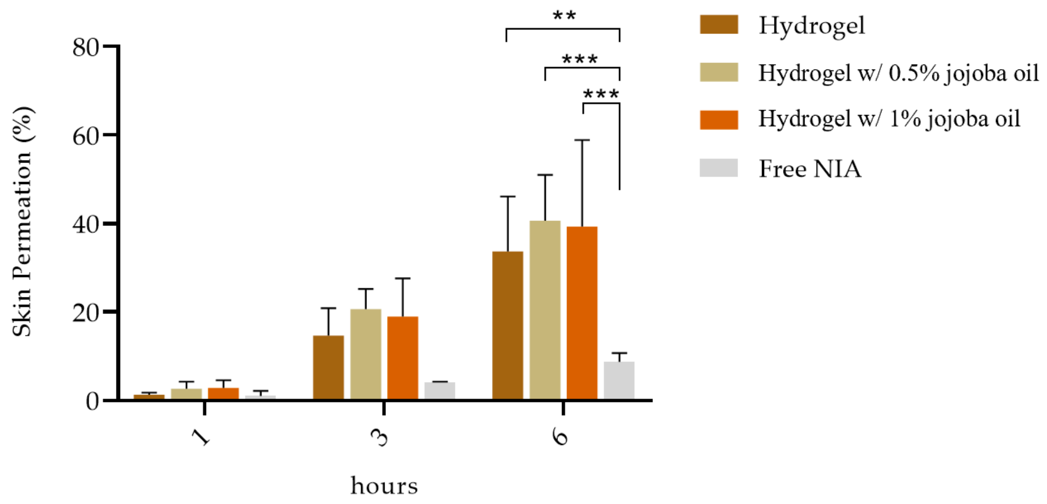

Figure S5. In vitro skin permeation results of free NIA, and NIA-loaded developed hydrogels, from four independent experiments (n = 4). Asterisks indicate statistical significance (*, $p \leq 0.05$; **, $p \leq 0.01$; ***, $p \leq 0.001$; ****, $p \leq 0.0001$). No asterisks indicate no statistical significance ($p > 0.05$).

Table S1. Characterization of non-loaded TEs.

	**PS (nm)**	**PDI**	**ZP (mV)**
tween 80	132 ± 1	0.290 ± 0.002	−15 ± 3
oleic acid	320 ± 27	0.316 ± 0.011	−31 ± 1
jojoba oil	249 ± 4	0.250 ± 0.011	−19 ± 1

Each value represents the mean ± SD of 3 independent measurements (n = 3). PS—particular size; PDI—polydispersity index; ZP—zeta potential; LC—loading capacity.
